# Intramuscular vs. Intradermic Needle-Free Vaccination in Piglets: Relevance for Animal Welfare Based on an Aversion Learning Test and Vocalizations

**DOI:** 10.3389/fvets.2021.715260

**Published:** 2021-08-11

**Authors:** Antoni Dalmau, Almudena Sánchez-Matamoros, Jorge M. Molina, Aida Xercavins, Aranzazu Varvaró-Porter, Israel Muñoz, Xènia Moles, Berta Baulida, Emma Fàbrega, Antonio Velarde, Joaquim Pallisera, Alba Puigredon, Alexandra Contreras-Jodar

**Affiliations:** ^1^Animal Welfare Program, Institute of Agrifood Research and Technology, Barcelona, Spain; ^2^HIPRA, Girona, Spain

**Keywords:** animal welfare, aversion test, behavior, needle-free vaccination, pain indicators, piglets, PRRS, vocalizations

## Abstract

The aim of the present study was to compare intramuscular injection with a needle and intradermic needle-free vaccinations against porcine reproductive and respiratory syndrome (PRRS) in piglets at 28 days old by studying behavioral and physiological reactions. A total of 72 piglets divided into 2 sex-balanced batches were assessed. Within each batch, the piglets were divided into three treatments, which were Hipradermic (0.2 ml of UNISTRAIN® PRRS vaccine administered with an intradermic needle-free device), Intramuscular (IM, 2.0 ml of vaccine), and Control (not vaccinated). Before the vaccination, the piglets were trained to cross a 4-m-long raceway to perform an aversion learning test. The day of vaccination, the time taken to cross the raceway was registered for each piglet at different times: prior to the vaccination and 10 min, 2, 24, 48, and 72 h after the vaccination, to measure variations in these times as signs of aversion to the vaccination process. Vocalizations, as potential signs of pain, were recorded as well at the end of this raceway to analyze their frequency (Hz), duration, and level of pressure (dB) at the moment of vaccination. Salivary cortisol, as a sign of the HPA-axis activity, was assessed 10 min after the vaccination. In addition, activity budgets, local reaction to the vaccine, and serological titer were also considered in the study. Ten minutes after the vaccination, the IM piglets took longer (*p* < 0.001) to cross the raceway than did the Hipradermic and Control piglets. Vocalizations were significantly different between the three treatments: the Control piglets produced vocalizations with the lowest frequency (*p* < 0.001) and level of pressure (*p* < 0.001), and IM with the highest, with Hipradermic in a significant intermediate position (*p* < 0.001). Accordingly, the day of the vaccination, IM and Hipradermic animals were lying on the side of the vaccine administration a greater proportion of time than were the Control piglets (10, 11, and 6%, respectively; *p* = 0.027). Salivary cortisol was not significantly different between treatments. The serum titer of antibodies against the PRRS was higher (*p* < 0.001) in both vaccinated treatments in comparison to the Control piglets. It is concluded that the Hipradermic needle-free vaccination may result in a less aversive experience in piglets than did intramuscular vaccination.

## Introduction

Pigs might be vaccinated by several routes, for instance, orally, intranasally, intravenously, or delivered onto, into, under, or across the skin or into a muscle (epicutaneous, intradermal, subcutaneous, transdermal, and intramuscular, respectively). Needle-syringe devices have been the predominant method for vaccine and drug delivery for pigs, usually by means of intramuscular administration. However, this procedure has been considered as potentially painful (1), resulting in acute and long-term fear (2). In humans, alternative systems such as the needle-free intradermal injection have been demonstrated to cause less pain and stress (3, 4). Other advantages of the needle-free technology in vaccination are the prevention of broken needles, elimination of residual needle fragments in pork carcasses (5), reduction of transmission of infectious diseases between animals (6), faster than a conventional needle-syringe, likely to reduce injury at the injection site, when compared to conventional needles (7), the use of lower vaccine volume, as compared to intramuscular injection (8), greater antigen dispersion (9), elimination of accidental worker self-injections (10), and elimination of needle disposal. Using mechanical compression to force fluid through a small orifice, these devices produce a high-pressure stream 76–360 μm in diameter (compared to 810 μm for a 21-gauge needle) that penetrate skin and subcutaneous tissue ensuring a homogenous process where each animal is vaccinated at the selected tissue depth (11). In the case of the intradermal application of vaccines, it is important to adequately adjust the pressure and force of the needle-free device to ensure a deposit of the vaccine specifically at the dermis layer.

Previous studies in pigs compared intramuscular injection with intradermal injection in terms of behavioral and stress indicators in commercial farms. Behavioral indicators used included activity budgets of the animals after the administration of the vaccine, and presence, frequency, and intensity of vocalizations at the moment of the vaccination (12–14). However, the degree of aversion after vaccination has not been explored before. This additional information can be obtained carrying out an aversion test by comparing the pigs' reaction when either IM or intradermic is performed under the same controlled conditions. This is based on motivations, which can be positive [e.g., the motivation to consume a commodity or perform a behavior (appetitive)] or negative [e.g., the motivation to avoid a painful or frightening stimulus (aversive or defensive)] (15–17). In operational terms, the aversion tests describe the tendency to approach or avoid resources and stimuli (18) and depend on the valence, i.e., the attractiveness (positive valence) or averseness (negative valence) of a situation. These tests have many strengths when compared with other approaches. First, they allow animals to express their own priorities, showing the most direct insight into what is important to them (19, 20). Second, motivation tests are highly sensitive to differences between treatments (21). Finally, they can ascertain whether an aversive stimulus is severe enough to cause suffering (19, 20). On the other hand, the most used indicator of stress is the cortisol concentration (14, 22) in some biofluids such as blood or saliva. Compared with blood sampling, saliva sampling is considered to be a noninvasive and less stressful methodology valid to assess the hypothalamic–pituitary axis activity (23).

Currently, the porcine reproductive and respiratory syndrome (PRRS) is the disease reported to cause the highest economic impact in modern pig production worldwide (24). As this impact affects not only the breeding herd but also their offspring, currently and increasingly, one of the most important vaccines in piglets is vaccination against the PRRS. Some European consumers seek greater animal-friendly products, which are directly related to a higher welfare standard at the farm level (25). Therefore, alternative routes to the classic intramuscular vaccination have been developed, including the PRRS. Specifically, the use of intradermal vaccination against the PRRS has been confirmed as an alternative to the intramuscular route, showing similar humoral and cell-mediated immune responses (26). Furthermore, intradermal vaccination against the PRRS has shown a better response in some parameters related to the behavioral and stress indicators in pregnant sows (22). However, data concerning the behavioral and HPA axis, as well as the aversion generated by the different routes in piglets in an experimental trial, have not been published. The aim of the present study is to compare the application of a vaccination against the PRRS in piglets by two different routes of administration, intramuscular and intradermal needle-free, by studying the pain response by means of analyzing vocalizations, aversion response by means of an aversion test, physiological stress by means of the analysis of salivary cortisol, and behavioral changes by means of activity budgets.

## Methods

The experiment was approved by the Institutional Animal Care and Use Committee (IACUC) of IRTA and the Catalan Government, under Code 11026.

### Animals and Treatments

Seventy-two (Landrace × Largewhite) × Pietrain piglets, half of them intact males and the other half females, divided into two batches of 36 animals each, were vaccinated in the present study. The study of each batch was separated over a span of 35 days. Neither the piglets nor the sows had ever been vaccinated against the PRRS at the farm of origin. The piglets were distributed into three groups according to the vaccination treatment: (1) Intramuscular vaccination (IM) by means of a single intramuscular injection in the neck region with 2.0 ml of UNISTRAIN® using an individual, stainless-steel needle of 21 G x 16 mm; (2) Hipradermic vaccination by means of a single needle-free intradermal application with 0.2 ml of UNISTRAIN® in the neck region using the Hipradermic® device, which is a battery-powered injector; and (3) Control treatment without vaccination and where the piglets were just touched in the neck region following the same handling procedure as the pigs in the previous groups. Vaccination occurred when the piglets were 28 days old. UNISTRAIN® PRRS (HIPRA, Amer, Spain) is a modified PRRS live vaccine that consists of an attenuated PRRSV1 strain (VP-046 BIS 10^3.5−5.5^ CCID_50_ per dose) diluted in PBS. This vaccine is authorized for piglets and sows for use by the intramuscular or intradermal route. In each case (intramuscular or intradermal route), the vaccine was prepared and used following the recommendations of the manufacturer. Before vaccination, these 72 piglets were selected from a total of 20 litters. Steps of piglet selection procedure and management were as follows.

After farrowing, all piglets born in crates of 10 sows per batch were weighed and arbitrarily ear-tagged with one of three possible colors: blue (Control), green (IM), or orange (Hipradermic) for treatment group distribution. In addition, each ear tag had a single number to allow for individual identification. The body weight of the piglets at birth from the first and second batches was 1.5 ± 0.30 kg and 1.5 ± 0.26 kg, respectively, with no significant difference between the different colors. No later than 24 h after birth, the farmer performed all the management procedures on the animals that potentially cause pain, such as iron application, teeth grinding, and tail docking, in order to concentrate all the aversive stimuli the first hours of life and avoid any other negative stimulus until the day of the vaccination. Tail docking was performed because, according to the farmer of the farm where the piglets were born and tested, he had some episodes of tail biting in the past, and the transportation to IRTA's research center (IRTA Monells, Girona, Spain) and change of environment at the age of 21 days could be an important risk factor for the appearance of this redirected behavior. Therefore, it was decided to allow the tail docking but leaving two-thirds of the tail. During lactation, some ear, but no tail, biting was observed. The iron injection was performed in the ham to leave the neck free for the PRRS vaccination. At 11 days of age, the piglets were socialized to optimize a human–animal relationship and maximize the piglets' comfort toward humans before the day of the vaccination. This socialization was performed by two researchers every day until the transportation in both batches (i.e., days 11, 12, 14, 15, 18, 19, and 20 of age). It consisted of gently approaching the animals to check their behavior toward human presence and trained to be milk-bottle-fed. This was important to facilitate the human–animal relationship at the arrival at the farm of destination and for the aversion test explained later. After day 15, 5 out of the 10 litters initially selected per batch were discarded for the study. The criteria to discard a litter was lack of uniformity, fearful reaction of the sow or piglets to humans despite socialization, lack of at least three good candidates of each treatment in terms of good health and body condition, or too imbalanced group in relation to sex (males and females).

When the piglets were 20 days old, and following the same criteria, only nine animals per litter (*n* = 45) were selected according to the same aforementioned criteria and individually weighed (Control: 5.6 ± 1.44 kg; IM: 5.9 ± 1.16 kg; Hipradermic: 5.8 ± 1.18 kg). Each group was equally composed of males and females. Afterward, at 21 days old, the piglets were transported for 3 h (200 km) from the farm of origin to the IRTA's research center where the study was carried out. The piglets were allocated at the truck into different compartments according to the litter to avoid mixing and fights. These groups of origin were also maintained at the destination and during the whole study for the same reason. Four of these five litters within each batch were selected as the main groups for the study, while the fifth was considered a reserve group, although these animals were trained and managed exactly in the same way as the other four. After arrival, animals were allocated to five pens of 2.80 × 2.40 m (6.72 m^2^), with a 100% plastic-slatted floor, three different types of enrichment material (balls, plastics, and ropes), two independent feeders with space for at least two piglets each, and two drinkers. On the day of arrival at the IRTA's research center, the pigs received the same handling procedure, including milk-bottle feeding with a milk replacer, from the same two researchers of IRTA who socialized the piglets at the farm of origin. This was done to reduce the piglets' stress and prevent abnormal behavior, such as tail or ear biting, which could cause painful injuries.

### Aversion Test

**Figure 1:**
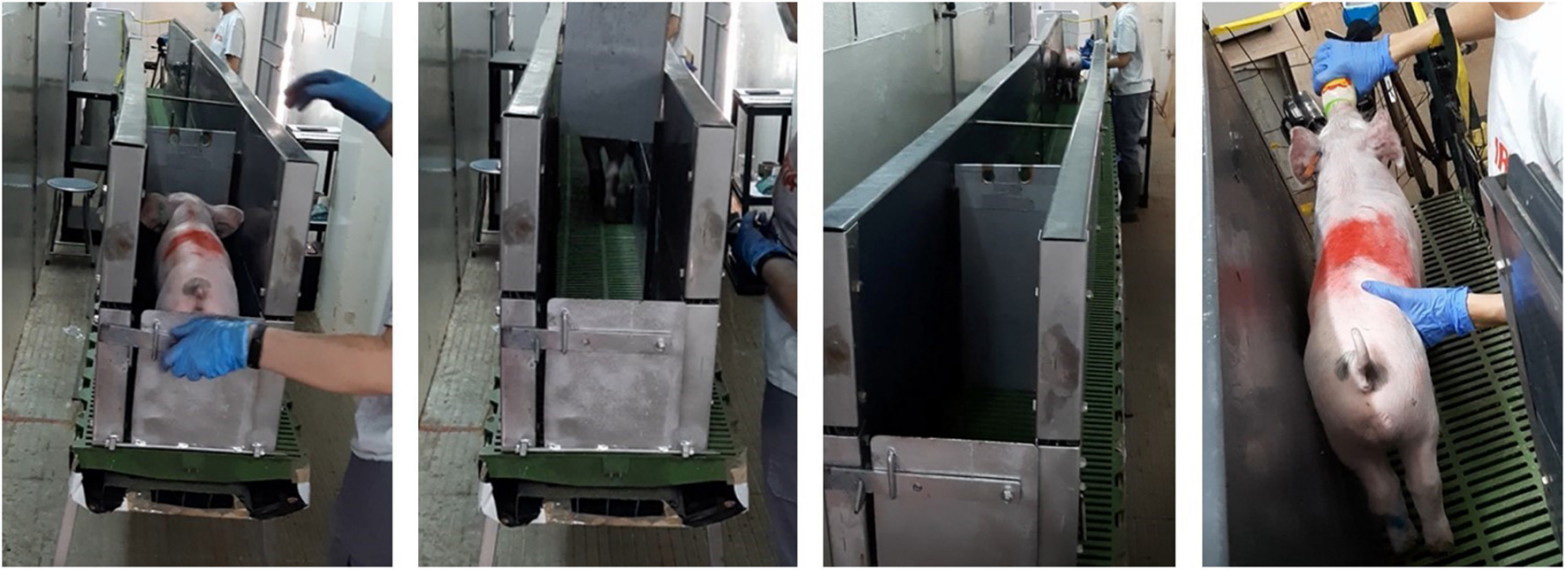
Raceway of 4 m long and 30 cm in width, elevated 1.2 m from the floor used for the aversion learning test. After a total of 14 training sessions, 72 piglets crossed this raceway until the end, and at this point, 24 were vaccinated intramuscularly, 24 with an intradermal needle-free system, and 24 not vaccinated. After this experience, the test was repeated, and the times recorded used as signs of aversion to the vaccination process.

The aversion test was carried out in a 4-m-long by 20-cm-wide internal raceway elevated 1.2 m above the ground (Figure 1). It was protected by a 40-cm-high by 5-cm-thick solid wall on both sides. The floor was 100% plastic slat like the pens' floors, but situated over a black base to avoid fearfulness of animals at perceiving the height to the ground. Animals were individually caught with both arms by the researcher and moved from the pens to the raceway. The distance from the pens of origin to the starting point of the raceway was less than 5 m in an independent building. From the first day, the same researcher was responsible for catching and releasing the piglets. During the two first training sessions, animals were gently encouraged and helped to walk along the 4 m of the raceway. At the end of the raceway, a second researcher, always the same, recorded the time to cross the raceway and fed the piglets with a milk replacer for a few seconds as a positive reward.

After four training sessions, those animals that took more than 2 min to cross the raceway were replaced by animals of the fifth pen. This happened with two animals from the first and one animal from the second batch. From the fifth training session onward, all the piglets were also gently touched on the right side of the neck before offering the milk. This procedure was always performed by the same researcher who also performed the vaccination. After 12 training sessions, the average time taken to cross the raceway was below 5 s, so animals that took longer than 10 s were discarded and replaced by animals from the fifth pen. This occurred with two more piglets of the first and one piglet of the second batch. Overall, from the 90 animals trained, only 6 (6.7%) were discarded for not being considered properly trained after 12 training sessions. In total, 14 training sessions were carried out from day 22 to day 27 of age. This means a maximum of three training sessions in a day and a minimum of one, with at least 1 h of resting between trainings. The piglets were vaccinated at day 28 of age.

On the day of vaccination, only 36 animals (12 per treatment) were included in the study. As in previous days, animals were moved to the raceway, but this time, IM and Hipradermic animals received the vaccination at the end of the raceway (by the same researcher as during the training sessions, at the right side of the neck, taking less than 3 s from the arrival of the animal to finishing the vaccination) before the milk replacer, while the Control piglets were firmly touched on the neck (trying to due an equivalent pressure to the vaccination) and provided milk. Each piglet repeated the test at 10 min, 2, 24, 48, and 72 h after vaccination, but this time instead of being vaccinated, they were inspected for the presence of any sign of reaction toward vaccination on the skin. In all cases, from the first training to 72 h after vaccination, the order of the piglets to run the test was the same, starting from Pen 1 and alternating one blue, one orange, and one green ear-tagged piglet successively until Pen 5.

### Vocalizations

Vocalizations were recorded just after vaccination (first 5 s from the start of procedure) with a digital voice recorder (Olympus VN-712 PC, Olympus; Tokyo, Japan) placed at 30 cm after the end of the raceway, where a sound-level meter (PeakTech 8005, PeakTech; Ahrensburg, Germany) was, as well, placed. The sound-level meter was used to record the maximum pressure of sound (energy, dB) emitted by the piglets at the moment of vaccination by using the C weighting that is ~80–90 phon. Vocalizations registered with the voice recorder were characterized according to their duration and the frequency (Hz) of the sound with the highest amplitude, known as peak frequency. According to Puppe et al. (27), they were classified in vocalizations of high (1,000 Hz or more) and low peak frequency (<1,000 Hz). For each recording, a researcher manually determined the start and end of each vocalization. A pause in the vocalization, usually associated with respiratory movements, was considered as a different vocalization according to Puppe et al. (27). Duration of each vocalization was assessed. The signal was filtered to eliminate any noise below 100 Hz, and each segment related to a vocalization was treated with a Hamming window to minimize possible problems at the beginning and the end of the signal. Afterward, the Fourier transformation was carried out. A single power analysis of the signal recorded for each vocalization was conducted, and the frequency with the highest power was selected as the peak frequency. Matlab 2008b was used for filtering and the Fourier analysis of each recording previous to the statistical analysis (28).

### Salivary Cortisol

The 72 piglets used in the study were sampled for cortisol analysis 24 h before and 10 min after the vaccination. Saliva samples from each piglet were collected on synthetic swabs (Salivette® Cortisol, Sarstedt; Nümbrecht, Germany). The swabs were fixed with forceps and placed around the back teeth for a maximum of 4 min to stimulate chewing. Then, Salivettes® were immediately centrifuged during 10 min at 3,500 rpm and the extracted saliva samples stored at −20°C until analysis of cortisol.

Salivary cortisol was analyzed using an automated chemiluminescent immunoassay validated for pigs (29).

### General Activity of the Animals

The general activity of the animals was assessed by means of scan- and focal-sampling methodologies the day of the vaccination, and the day before and after by means of direct observations carried out by two trained observers. As vaccination was carried out during the morning, the general activity was assessed from 16:00 to 20:00 h on all 3 days. Ten minutes before starting the observations, the observers entered the room and walked around to allow the piglets to get used to their presence. Then, animals from each pen were scan-sampled at 10-min intervals, and the number of pigs engaged in the different behavior categories described in Table 1 was recorded. Therefore, a total of 5,400 observations resulted from 150 scans (25 scans per day × 3 days × 2 batches) of 36 animals. In addition, focal sampling was carried out by direct observation of each animal for 4 min. Thus, from 16:00 to 17:00 h, the piglets of Pen 1 were observed, from 17:00 to 18:00 h of Pen 2, from 18:00 to 19:00 h of Pen 3, and from 19:00 to 20:00 h of Pen 4. In Pen 5, only the piglets used as replacement of the discarded animals from Pens 1 to 4 were assessed. The same behavioral categories as for scan samplings were used, but social behavior was classified into two categories according to Welfare Quality® (30): negative social behavior (the receiver of an action was reacting negatively to the contact by flight or fight) and positive social behavior (the receiver did not react negatively to the contact). The observers were both blinded to the treatment, as they were not present during the vaccination. They performed a training session with the coordinator 3 days previous to the vaccination with a Kappa value of 0.83 in terms of interobserver repeatability. They were assigned to different pens, so Pens 1 and 3 were for observer A and Pens 2 and 4 for observer B; Pen 5 was one day for observer A and one day for observer B. Inside each pen, there were three piglets from each treatment, so all treatments were balanced. While observer A was doing focal sampling of Pen 1, observer B was doing scan samplings of all pens at once (at 10-min interval). While observer B was doing focal samplings of Pen 2, observer A was doing scan samplings of all pens at once.

**Table 1:** Activities assessed during the scan sampling.

**Behavior**	**Definition**
Lying sternally	The piglet is not bearing weight on any of the limbs, and the major part of the sternal region is in contact with the floor
Lying on right side	The piglet is not bearing weight on any of the limbs, and the right side of its body is in contact with the floor (area of the vaccination).
Lying on the left side	The piglet is not bearing weight on any of the limbs, and the left side of its body is in contact with the floor (opposite area of the vaccination).
Scratching	Piglet scraping its body against the facilities of the pen.
Exploring	The piglet licks with its tongue or touches with its nose or sniffs to less than 5 cm any part of the pen, except the internal part of the drinker and the feeder.
Social	Mouth or nose of a piglet in contact with the body of another one.
Eating	Piglet eating or exploring feed, that means with food in its mouth.
Walking	Piglet just walking (using their legs to travel distances) and not performing any of the previous behaviors.
Sitting	Piglet resting on their hindquarters in a dog sitting position without performing any of the previous behaviors.
Standing	Piglet standing, using all its four legs, but in a static position, not moving and not performing any of the previous behaviors.
Other	Any piglet in an attitude impossible to classify in any of the previous descriptors.

### Reaction to the Vaccine

The area of vaccination (the right side of the piglet's neck) was assessed for the presence of papule, inflamed area, redness area, ulcer, crusted area, nodule, and blood spot. The development of skin condition and potential reactions to vaccination were tracked from a few seconds prior to the vaccination to 10 min, 2, 8, 24, 48, 72 h, and 21 days after the vaccination. Only those reactions equal to or bigger than 1 mm were recorded.

### Immunity

Animals were maintained until day 21 after vaccination, and then a blood sample was taken for analysis of antibodies against the PRRS. Sera were tested using a commercial ELISA kit for the presence of specific antibodies against PRRSV (indirect ELISA IDEXX PRRS X3 Ab Test: IDEXX Laboratories). According to the manufacturers, a sample with an S/P ratio greater than 0.4 was considered to be positive for the PRRS ELISA.

### Statistical Analysis

Analyses were carried out with the Statistical Analysis System (SAS software, SAS Institute Inc.; Cary, NC, USA). Normality of residuals was checked through the Shapiro–Wilk test and QQ plots of residuals for each one of the dependent variables studied. When a variable met the normality assumption, a linear model by means of Proc Mixed was used. Data from the aversion test were analyzed by repeated measures with treatment (IM, Hipradermic, and Control) and time of testing as fixed effects (less than 10 s prior to vaccination, 10 min, 2, 24, 48, and 72 h after vaccination) and its interaction including the animal as the random effect. The maximum pressure of sound from the piglets' vocalizations was assessed by means of Proc Mixed to compare values between treatments, and a binomial distribution to ascertain the presence or absence of vocalizations above 90 dB was used. The number of vocalizations per animal (low and high frequencies and total) was analyzed by means of Poisson or negative binomial distribution according to Cameron and Trivedi (31). Salivary cortisol was analyzed using a Poisson distribution considering a vaccination treatment and time points (prior to and 10 min after vaccination) as fixed effects. In the case of scan sampling, each behavior (resting, lying sternally, lying on right side, lying on left side, scratching, exploring, social, eating, walking, standing) was analyzed separately by means of binomial distribution and considering the treatment, the different time points (day before vaccination, day of vaccination, and day after vaccination), and observer as fixed effects and the pen as a random effect. Focal sampling was transformed to percentage of time out of the total time observed with the animal performing a specific activity and analyzed using a Poisson or negative binomial distribution according to the deviance for each one of the behaviors assessed and considering the vaccination treatment, time points (day before vaccination, day of vaccination, and day after vaccination), and the observer as fixed effects and the pen as a random effect. The reaction to the vaccine was analyzed by means of a binomial distribution (presence/absence) including the vaccination treatment and the different time points (less than 5 s after the vaccination and 10 min, 2, 8, 24, 48, and 72 h after vaccination). Finally, the antibody titer was analyzed by means of a Proc Mixed analysis. When possible, and when significant differences were found, the least-square means of fixed effects (LSMEANS) was used for multiple comparisons. In all cases, significance was fixed at *p* < 0.05.

## Results

### Aversion Test

**Figure 2:**
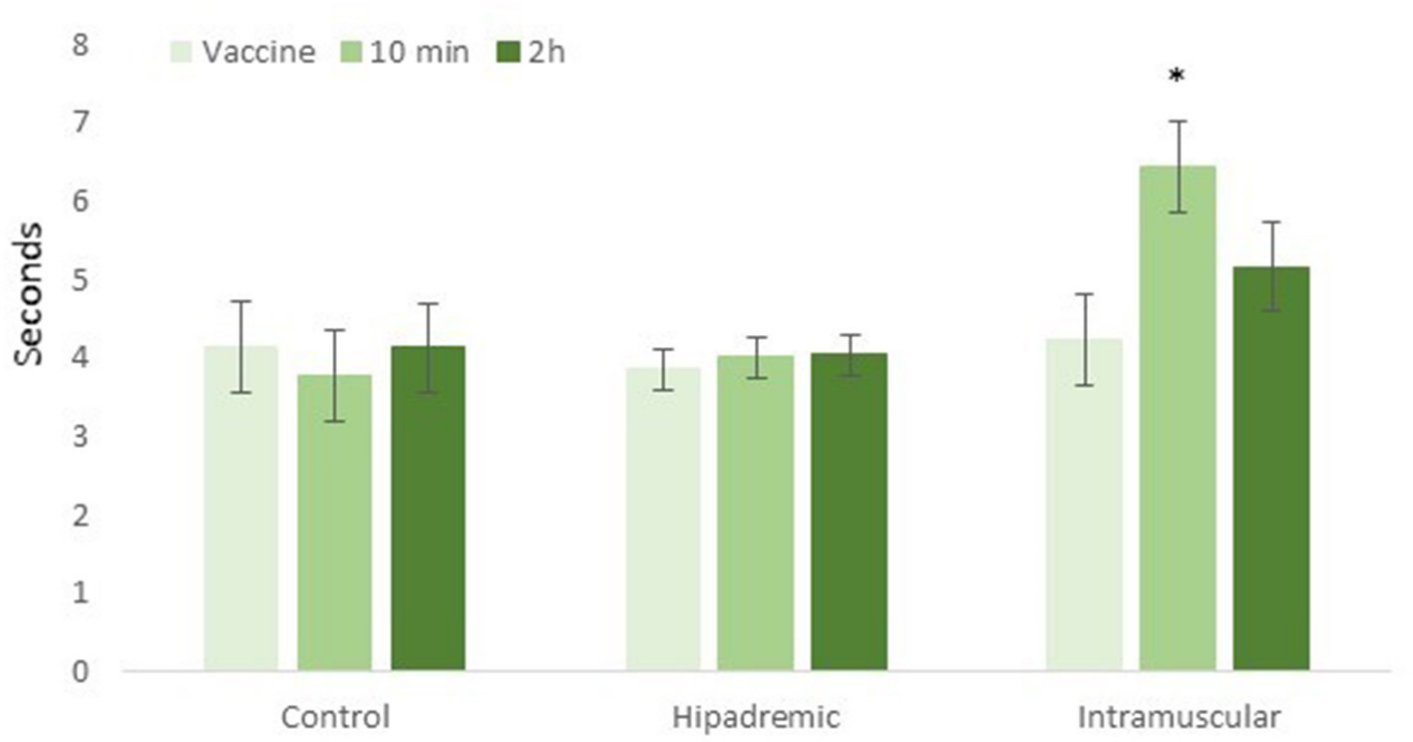
Time taken for the piglets to cross the 4-m-long raceway just prior to the vaccination (vaccine), 10 min after the vaccination (10 min), and 2 h after the vaccination (2 h) for the three treatments: Control, Hipradermic, and Intramuscular. *means significant differences at *p* < 0.001.

Before the vaccination (time 0), the time taken for the animals to cross the 4 m of the raceway was 4.2 ± 0.58 s, 3.9 ± 0.26 s, and 4.2 ± 0.58 s in the Control, Hipradermic, and IM groups, respectively. This time was not significantly different between treatments (*p* = 0.8022) and only increased significantly in relation to the basal during the test performed 10 min after the vaccination in the IM treatment group (*p* < 0.001), reaching a time of 6.5 ± 0.58 s, and being significantly higher (*p* < 0.001) than the other two treatments (Figure 2). Two hours after the vaccination, the difference was again not significant between treatments (*p* = 0.1319), with a significant decrease (*p* < 0.001) in the IM treatment, in comparison to the time recorded 10 min after the vaccination (Figure 2). One day, two days, and three days after the vaccination, no differences were found between treatments or with the values obtained on day 0 (*p* > 0.05), with a mean time of 4.8 ± 0.37 s, 4.6 ± 0.44 s, and 4.9 ± 0.59 s in crossing the raceway for Control, Hipradermic, and IM, respectively.

### Vocalizations

**Figure 3:**
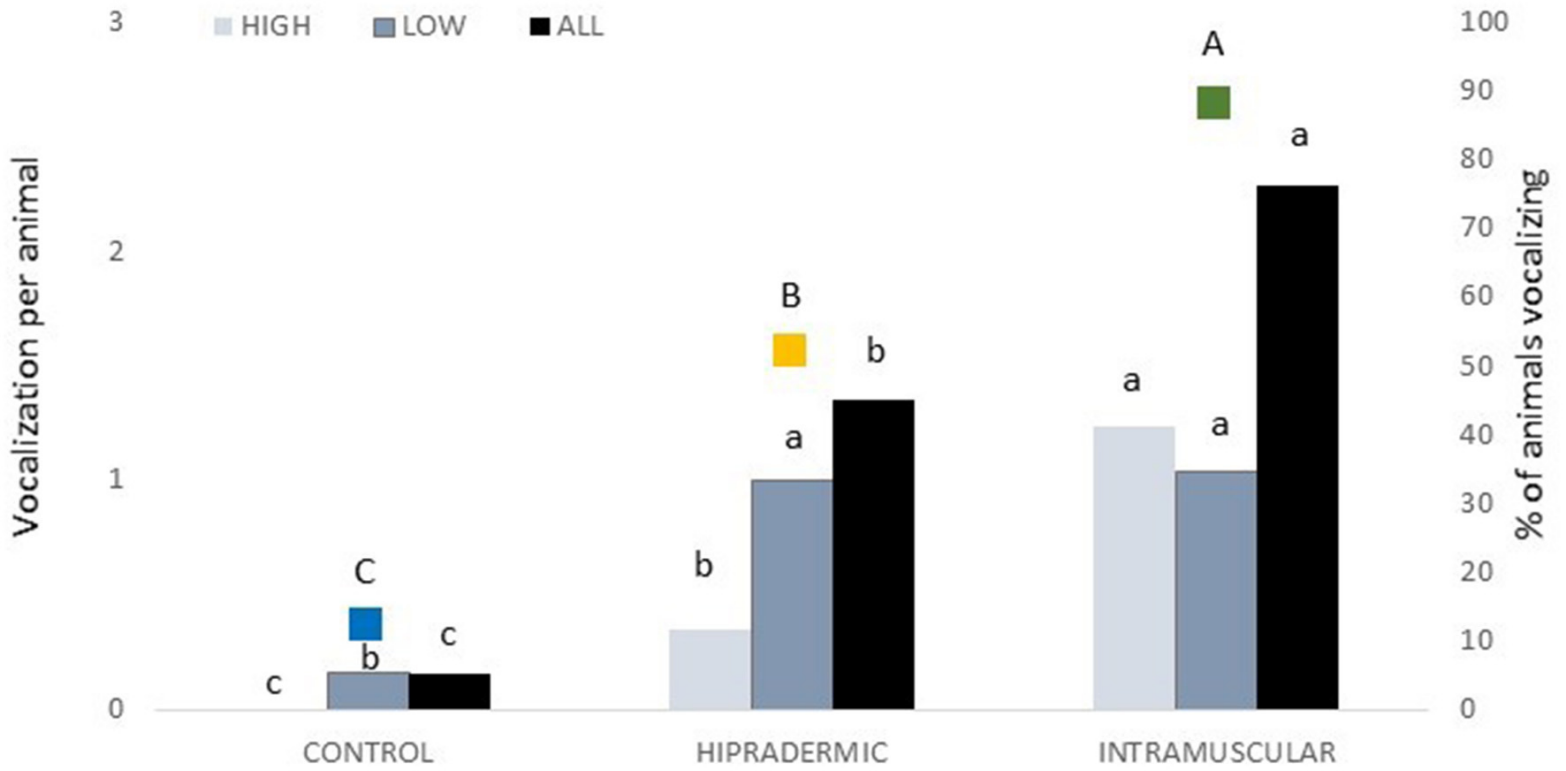
In bars and associated with the left axis, the number of high peak (1,000 Hz or more), low peak (<1,000 Hz), and the total peak frequency vocalizations per treatment (Control, Hipradermic, and Intramuscular). In colored squares and associated with the right axis, the percentage of animals vocalizing at the moment of vaccination per treatment (blue: Control; Orange: Hipradermic; and Green: IM). Different lowercase letters mean significant differences within treatment in frequencies of high and low vocalizations, and different capital letters mean significant differences between treatments in % of piglets vocalizing at *p* < 0.05.

A total of 93 vocalizations were registered during the vaccination corresponding to 5 in Control, 31 in Hipradermic, and 57 in IM treatment, as the same animal could have more than one vocalization during the vaccination. The maximum number of vocalizations was found in one piglet with 10 consecutive vocalizations. Duration ranged from 104 to 1,416 ms, and no statistical differences (*p* > 0.05) were found among treatments (515 ± 118.2 ms, 406 ± 27.4 ms, and 459 ± 37.6 ms in Control, Hipradermic, and IM, respectively). Nevertheless, a treatment effect (*p* < 0.001) was found in the percentage of animals vocalizing (*p* < 0.0001) and in the number of vocalizations performed per animal (*p* < 0.001). In the Control group, only three animals vocalized (12% of the total), being significantly less (*p* < 0.01) than IM and Hipradermic treatments (Figure 3). In addition, in the Hipradermic treatment, the percentage of animals vocalizing (52%) was significantly lower as well (*p* = 0.010) than in IM (88%; Figure 3). The number of vocalizations per animal was lower (*p* < 0.01) in the Control piglets (0.16 ± 0.096 vocalization per animal) than in the other two treatments, and lower (*p* = 0.0306) as well in Hipradermic (1.35 ± 0.372 vocalizations per animal) than in IM (2.28 ± 0.414 vocalizations per animal; Figure 3). When classified in vocalizations of high (1,000 Hz or more) and low peak frequency (<1,000 Hz; 27), 41 were high and 52 low. In the Control piglets, no high-frequency vocalizations were observed, and a significant effect (*p* = 0.0182) was found in the number of these vocalizations for the Hipradermic piglets (0.35 ± 0.132 high peak frequency vocalization per animal) in comparison to IM (1.24 ± 0.290 high peak frequency vocalizations per animal; Figure 3). When the low peak frequency vocalizations were considered, differences (*p* < 0.05) between the Control, with 0.16 ± 0.096 low peak vocalization per animal, and the other two treatments (with 1.00 ± 0.308 and 1.04 ± 0.336 low peak frequency vocalizations per animal in Hipradermic and IM treatments, respectively) were found, being less frequent in the Control than in the other two treatments (Figure 3).

**Figure 4:**
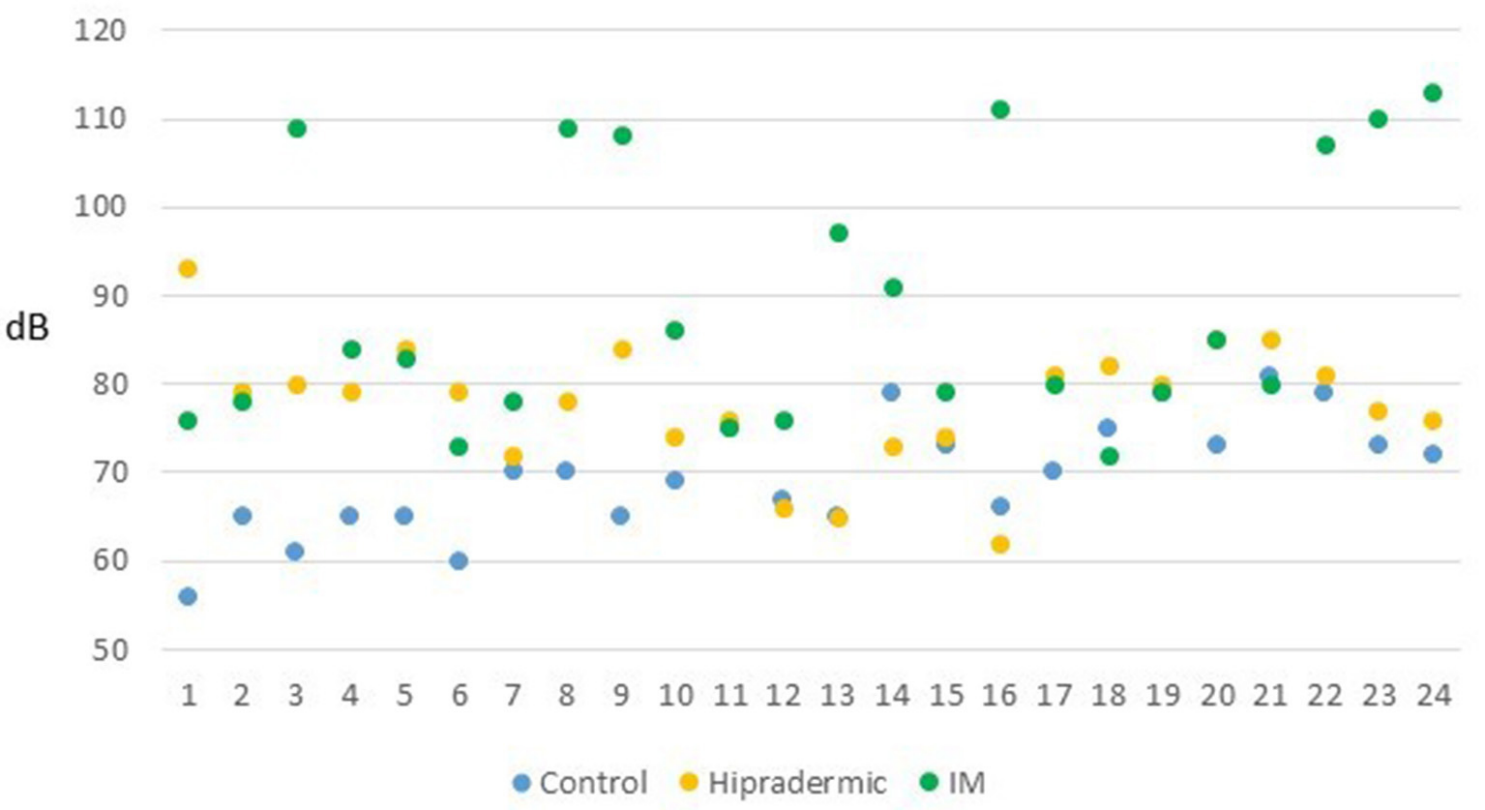
Maximum level of pressure of sound achieved by the 24 animals (dB) of each treatment (blue: Control group; orange: Hipradermic; and green: IM) at the moment of the vaccination measured with a sound meter at 30 cm of distance from the face of the animal.

The level of pressure of sound achieved by the animals, assessed in dB, with the sound level meter at 30 cm from the face of the piglet at the moment of the vaccination, was affected by the treatment (*p* < 0.001). The mean value in the Control treatment was lower (70 ± 1.9 dB) than in the other two treatments, and Hipradermic was lower (78 ± 1.9 dB) than was IM (89 ± 1.9 dB). In fact, the percentage of animals that achieved 90 and 100 dB was significantly lower (*p* < 0.05) in Control and Hipradermic groups than in IM (Figure 4).

### Salivary Cortisol

No treatment effect during the basal sampling or the sampling taken 10 min after vaccination was found for salivary cortisol (*p* > 0.05). Ten minutes after vaccination, the salivary concentration was 0.125 ± 0.0118 ng, 0.114 ± 0.0059 ng, and 0.134 ± 0.0086 ng of cortisol/g saliva for Control, Hipradermic, and IM groups, respectively.

### General Activity

**Figure 5:**
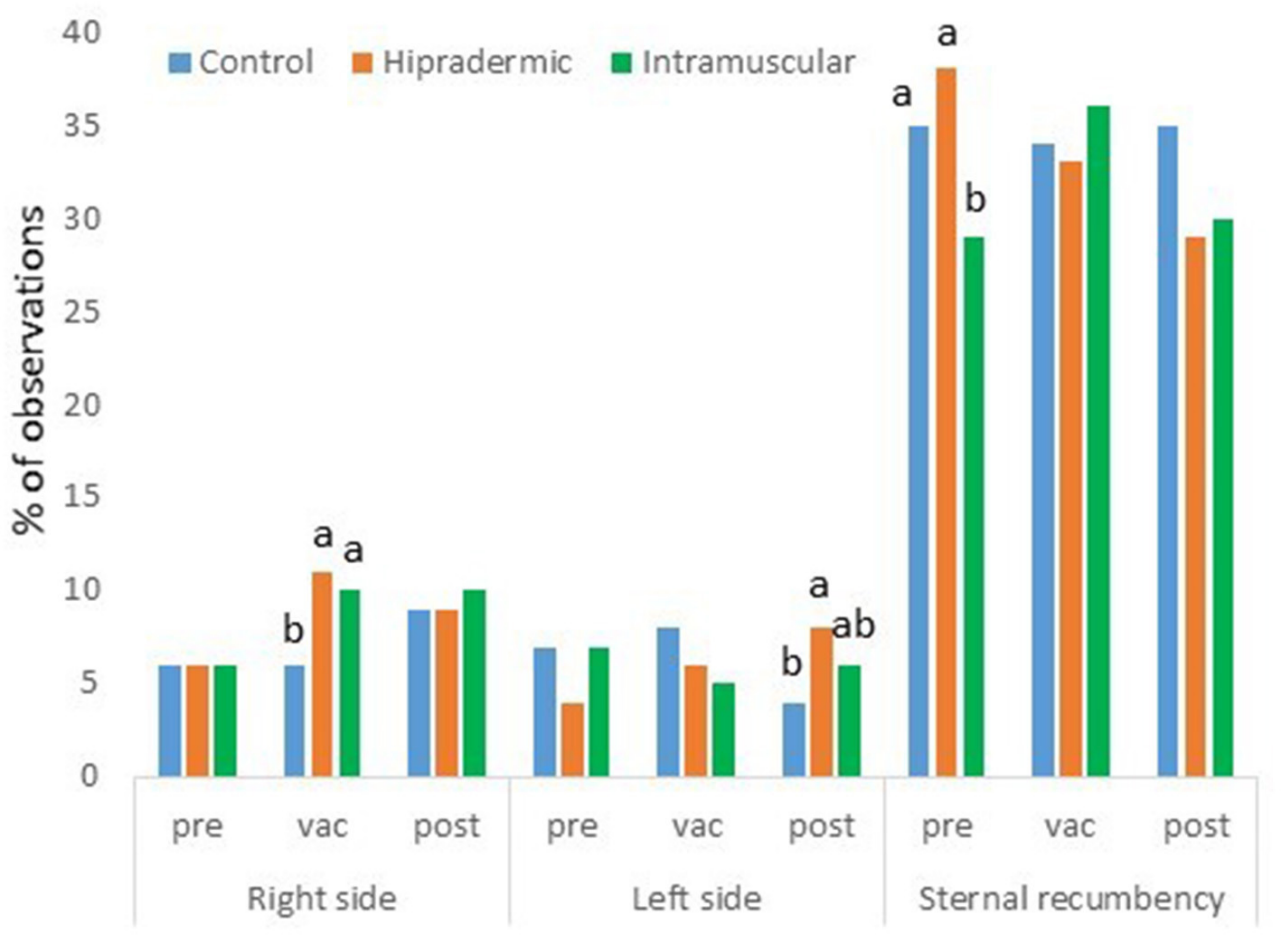
Percentage of the observations where piglets were seen resting on their right side, the left side, or in sternal recumbency for treatment (blue: Control; orange: Hipradermic; and green: IM), the day prior to the vaccination (pre), the day of the vaccination (vac), and the day after the vaccination (post). Different letters mean significant differences at *p* < 0.05.

According to the scan sampling, the main activity the day prior to the vaccination, the day of the vaccination, and the day after the vaccination was resting, ranging from 43 to 49% of the observations, and with no treatment effect in any of the days assessed. During resting, three different positions were assessed: sternal recumbency, lying on their right side, and lying on their left side. Sternal recumbency was affected by the treatment (*p* = 0.0136) the day previous to vaccination. Animals from the IM group lay less frequently in this position (29 ± 2.1% of the observations) than did Hipradermic animals (38 ± 2.4% of the observations; Figure 5). This difference was not observed the day of the vaccination nor the day after the vaccination, with only a more frequent trend (*p* = 0.0836) to rest by sternal recumbency in Control animals (35 ± 2.2%) than in Hipradermic (29 ± 2.0%) and IM treatments (30 ± 2.5%). The day of the vaccination, a treatment effect was found for animals lying on their right side (*p* = 0.0272). The piglets of the Control group spent less time in this position (6 ± 0.9%) than did Hipradermic (11 ± 1.2%) and IM (10 ± 1.1%) animals (Figure 5). In addition, the day after the vaccination, a treatment effect was found for animals lying on their left side (*p* = 0.0312). The piglets of the Control group spent less time in this position (4 ± 1.1%) than did Hipradermic animals (8 ± 1.3%), the IM group being in between and with no significant differences in relation to the other treatments (6 ± 1.2%; Figure 5).

The second-most common activity after resting was eating, ranging from 11 to 18% of the observations and with no differences between treatments in any of the 3 days assessed. Similar numbers of animals were found to be exploring, ranging from 11 to 16% of the observations and with just a trend in differences between the treatment groups on the day of the vaccination (*p* = 0.0871), with the Control animals showing the tendency to explore more (16 ± 2.3% of the observations) than did the IM group (11 ± 1.7% of the observations), and the Hiprademic having intermediate values (13 ± 2.1% of the observations). The next activity in terms of percentage was social behavior, ranging from 9 to 14% of the observations. In this activity, no treatment effect (*p* > 0.05) was found. The piglets were seen walking between 2 and 6% of the observations, standing in 1–5% of the observations, sitting in 0–2% of the observations, and scratching in 0–1% of the observations, with no treatment effect for any of those behaviors on any of the observation days (*p* > 0.05).

Regarding focal sampling, the piglets rested during 47 ± 2.9%, explored during 14 ± 1.3%, and ate during 12 ± 1.9% of the time. Evaluation of social behavior by focal sampling was divided as previously explained in Section 2.5, with positive being 9 ± 1.1% of the total time of observation and negative being 4.6 ± 0.72%. Walking was recorded during 5 ± 0.5% and standing 3.8 ± 0.36% of the time. None of these behaviors showed a significant treatment effect in any of the days assessed. However, sitting, which lasted 0.8 ± 0.15% of the time, and scratching, with 0.2 ± 0.06% of the time, showed a significant difference (*p* < 0.05) among treatments. In fact, the day of the vaccination (*p* = 0.0145), the piglets from the IM group spent less time sitting (0.04 ± 0.041% of the time) than did the Control (1.2 ± 0.51% of the time) and Hipradermic (1.2 ± 0.70% of the time) piglets. The same day (*p* = 0.0081), the piglets from the Hiprademic group spent less time scratching (0.04 ± 0.089% of the time) than did the Control (0.26 ± 0.119%) and IM (0.46 ± 0.2582%) piglets. Finally, the day after the vaccination (*p* = 0.0002), the piglets from the IM group spent less time scratching (0.08 ± 0.076%) than did the Control (0.33 ± 0.154%) and Hipradermic (0.33 ± 0.146%) piglets.

### Reaction to the Vaccine

The statistical analysis of this section did not consider the Control group, as nothing was seen, nor expected, in these animals. In addition, none of the Hipradermic or IM piglets had any reaction in the skin prior to the vaccination. In contrast, the presence of a blood spot was only seen in IM treatment, being significantly higher just after the vaccination (*n* = 4, 17%, *p* = 0.0151) and 10 min after the vaccination (*n* = 7, 29%, *p* = 0.0076), in comparison to the Hipradermic piglets (0% in both cases).

### Immunity

**Figure 6:**
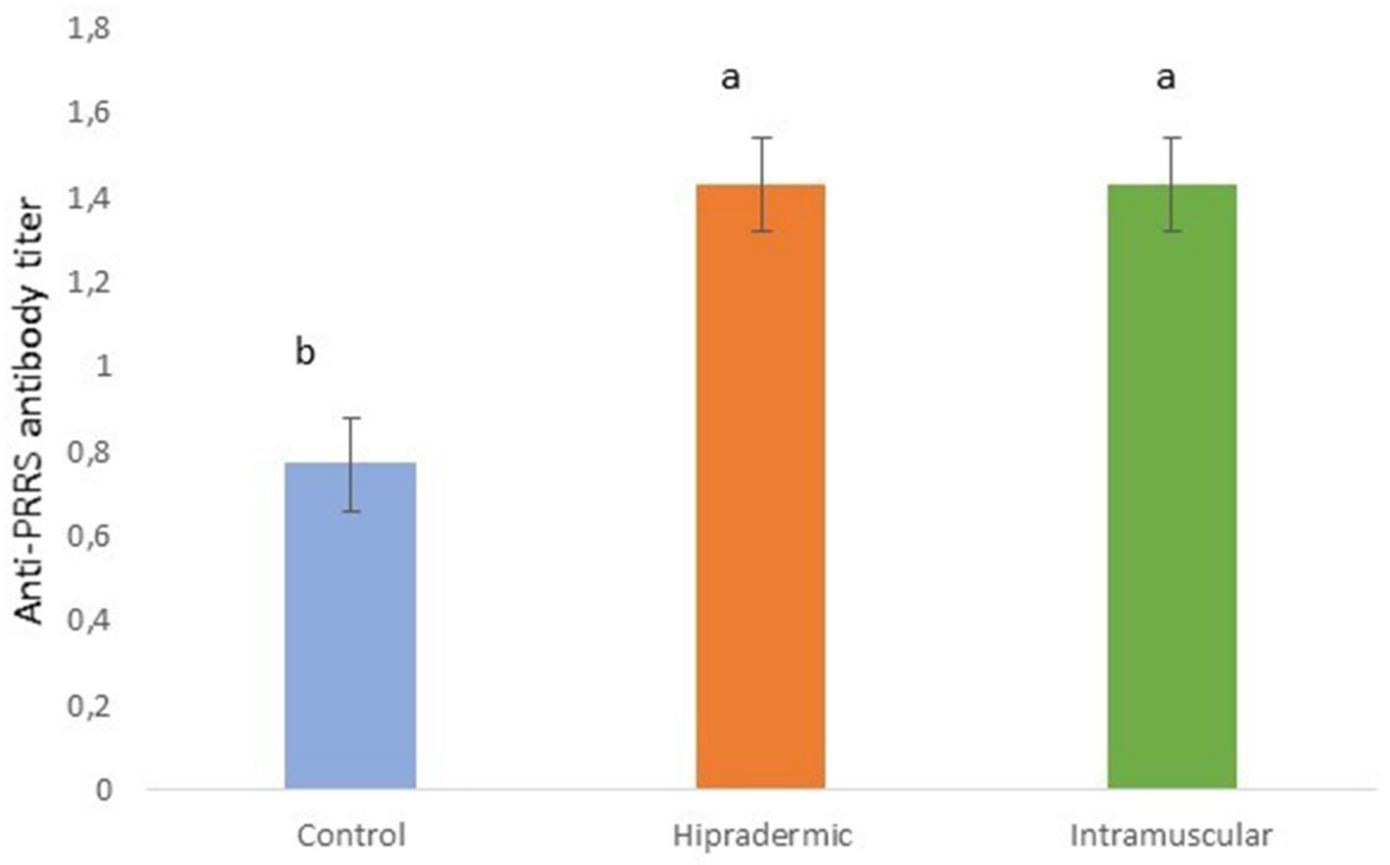
Serum titer of antibodies against the PRRS in piglets 50 days old, 21 days after the Hipradermic, Intramuscular vaccination, and no vaccination (Control) with a life-attenuated virus vaccine (UNISTRAIN®). Different letters mean significant differences at *p* < 0.05.

All the piglets of the study showed some antibody titer 21 days after the vaccination. In the case of the Control animals, which were not vaccinated, the titer was significantly lower (*p* < 0.001) than in the Hipradermic and IM animals (Figure 6), the last two not being significantly different between them. In addition, 52% of the animals from the Control group achieved an S/P ratio higher than 0.4, considering them as seropositive.

## Discussion

The overall results of this study show that the piglets vaccinated with the same product against the PRRS virus, but by two different routes of administration, Hipradermal needle-free vs. intramuscular injection with a needle, had a similar immune response, as reported previously by Madapong et al. (26), but differed in the occurrence of some signs of pain and aversion. A commercial vaccine against the PRRS virus (UNISTRAIN®) that contains a life-attenuated virus, free of any adjuvant, was selected. Therefore, the results of the present study should be considered just under this scenario, as an adjuvant could produce a different pain response. In addition, it is important to consider that in the case of the Hipradermal administration, only 0.2 ml of liquid was used, while in the intramuscular administration, it was 2.0 ml, since it is the required dose for the two types of administration. Finally, the present study was carried out under experimental conditions in a research center on animals trained to be in contact with humans and touched in the neck at the place of vaccination to isolate the effect of the vaccination from other potential stimuli as much as possible. In addition, for the aversion test, 6 out of 72 animals (8%) were replaced because they were considered not properly trained (they took more than 2 min after four training sessions or more than 10 s after 12 training sessions in crossing the raceway).

The piglets' response to the route of vaccine administration was assessed through behavioral, physiological, and clinical indicators (skin reactions), just at the moment of the administration and afterward. At the moment of administration, the behavioral indicator assessed was vocalizations. In piglets, vocalizations can be used as an indicator of pain (32, 33), although they are an unspecific indicator. In fact, Marx et al. (32) indicated that the pressure of sound (dB), frequency (Hz), and call duration were critical in analyzing pain-related vocalizations in these animals. In the present study, the percentage of vocalizations was lower in the Control group (not vaccinated) than in the Hipradermic and IM treatments. In addition, it was lower, too, in the Hipradermic than in the IM treatment. This agrees with the results presented by Scollo et al. (14) and Temple et al. (13) with regard to differences between intradermal and intramuscular administration, although Scollo et al. (14) did not use a Control group and Temple et al. (13) found no differences between the control and intradermal groups. Actually, the percentage of piglets that vocalized during the vaccination was slightly lower in Temple et al. (13), 7, 7, and 32% for the Control, intradermal, and intramuscular groups, respectively, as compared to Scollo et al. (14), who reported 45 and 75% for intradermal and intramuscular administrations, respectively, and to the present study, with 12, 52, and 88% for the Control, Hipradermic, and IM treatments, respectively. However, a reason for this difference could be that Temple et al. (13) considered just high-pitched vocalizations assessed by direct observation, and not recorded. In fact, high-frequency vocalizations, defined as higher than 1,000 Hz, are more associated with pain than are low frequencies (27, 34). Accordingly, when the vocalizations of the present study were analyzed, only 40% were above 1,000 Hz, and none of the animals in the Control group vocalized above these high frequencies. Nevertheless, again, the Hipradermic group showed less high-frequency vocalizations (0.35 per animal) than did the IM group (1.24 per animal), supporting a difference in pain perception.

Scollo et al. (14) reported, as well, that the pressure of sound achieved by the animals' calls was lower when using an intradermal route of administration rather than intramuscular (91 and 100 dB, respectively), similar to the results found in the present study as well (70, 78, and 89 dB for Control, Hipradermic, and IM, respectively). When the percentage of animals achieving 90 or 100 dB was considered, in both cases, there was a significant difference with a higher value in the IM treatment, in comparison to the Hipradermic (Figure 4). Regarding the duration of the vocalizations, although Scollo et al. (14) found a significant difference between intramuscular (588 ms) and intradermal (352 ms), in the present study, this difference was not found (515, 406, and 459 ms for Control, Hipradermic, and IM, respectively). Therefore, taking into consideration the presence of vocalizations and maximum pressure of sound, it could be concluded that piglets had more signs of aversion when vaccinated than when only handling was conducted, and more signs of aversion when vaccinated with an intramuscular injection than with a needle-free intradermal device. However, one of the main objectives of this paper, not tested before, was to ascertain if this negative experience could be detected by means of an aversion learning test. This type of test is useful to ascertain whether an aversive stimulus is severe enough to cause suffering (19, 20) and has been used in the past in pigs, for instance, to study the degree of aversion to different concentrations of carbon dioxide in atmospheric air (35). In this case, it is useful to ascertain at which level the vaccination is an aversive situation (negative valence) for the animal. In consequence, after a selection process and a proper training process in which the individuals became accustomed to the facilities, in this case a 4-m-long raceway, and considering that a positive reward was offered to them at the end (milk given in a feeder bottle), piglets had the opportunity to express their own priorities (approaching faster or slower) according to their experience during vaccination at 10 min, 2, 24, 48, and 72 h after the administration. According to the results, only in the group subjected to the intramuscular injection and only at 10 min after the vaccination did the time taken by the piglets in crossing the raceway increase. This confirms that intramuscular administration of a vaccine was the worst experience for the piglets in comparison to the other two treatments, with an increase of 1.5-fold time, similar to the results found in Dalmau et al. (35) for pigs after exposure to different concentrations of carbon dioxide. However, the fact that 2 h after the vaccination this difference disappeared indicates that the aversive stimulus had low severity for the animal.

The third indicator assessed was a physiological parameter: salivary cortisol. This indicator was used also in previous studies comparing intramuscular vs. intradermal vaccination (14, 22), and in agreement with the present study, no treatment effects were reported. In fact, this indicator has been used in other studies addressing the consequence of painful procedures in pigs, showing significant differences for procedures like castration and tail docking (36, 37). However, Scollo et al. (14) argued that the lack of differences in plasma cortisol between these two different routes of administration of a vaccine might suggest that this parameter might not be sensitive enough to recognize stress during apparently low painful interventions. Temple et al. (13) tried other indicators, such as salivary alpha-amylase (SAA) and salivary Chromogranin A (CgA), but they failed in showing differences between treatments as well. Therefore, it is possible that the animal could cope with the situation just with a behavioral response, with limited effects in a physiological response. On the other hand, although according to Buwalda et al. (38) changes in cortisol level can be observed 5 min after a stimulus, and samples in the present paper were taken after 10 min since the stimulus, it cannot be disputed that the physiological reaction on the animals could occur later and in consequence not detected.

In terms of behavioral response, in addition to those more associated with the vaccination (vocalizations at the moment of injection) or in the place of the vaccination (time in crossing the raceway after the injection), the activity budgets a few hours after the vaccination, and more than 24 h later, they have been studied in this and in previous studies as well. In the present study, no differences were found between treatments in the time spent resting (non-active), eating, performing negative or positive social behavior, standing, or exploring. Only the day of the vaccination, in the afternoon, a trend (*p* = 0.0871) of greater frequency of exploration was found in the control animals, as compared to the Hipradermic and IM piglets. In addition, two behaviors with a very low prevalence (sitting and scratching with the pen structures), representing <1% of the total activity performed by the animals, showed significant differences (*p* < 0.05). The day of the vaccination, animals from the IM treatment spent less time sitting than did the other two treatments. However, due to the low prevalence, the biological value is low. On the other hand, the day of the vaccination, the Hipradermic piglets reduced their scratching behavior, in comparison to the other treatments, and the day after the vaccination, the IM piglets performed this behavior during less time, in comparison to the other two treatments. Hay et al. (39) considered scratching the rump as a sign of pain after surgical castration, so a similar hypothesis could be suggested after vaccination, but in both cases, the day of the vaccine and the day after, the Control group behavior did not support this hypothesis. In fact, the prevalence of this behavior was less than 0.5% of the total activity registered; therefore, probably, any conclusion on this would be too speculative. Conversely, Temple et al. (13) found a decreased activity in intramuscularly injected piglets, in comparison to the intradermal group, including less exploratory and negative social behavior, whereas Göller et al. (12) found a faster recovery of the sucking behavior in unweaned piglets intradermally vaccinated than when they were injected intramuscularly. Nevertheless, these studies were performed with different adjuvanted vaccines. Adjuvants may enhance side-effects and nonspecific systemic reactions, such as fever or anorexia (40), and it could be related to the difference in the behavioral response between groups.

The day of the vaccination, piglets in both vaccination treatments were seen more frequently resting on their right side (the side of the vaccination) in comparison to the Control ones, which maintained similar values prior to the vaccination (Figure 5). The day after vaccination, the vaccinated animals tended to rest more time in a lateral position than did the Control animals, with a higher preference for the right side (the side of the vaccination) than the left side (especially in the case of IM treatment). This correlates with the reaction to the vaccination found in the skin. For instance, a papule in the Hipradermic and a blood spot in the intramuscular groups were seen up to 10 min after vaccination, but the presence of crusts and redness areas achieved a maximum in both treatments just at the end of the assessment of activity budgets the day of vaccination (8 h after vaccination) and were maintained in similar values the day after (30 and 58% of crust in Hipradermic and IM treatments, respectively, and 33–24% and 25–21% of redness areas the day of the vaccination–day after the vaccination in the Hipradermic and IM treatments, respectively). These results disagreed with those obtained by Temple et al. (13), where the piglets vaccinated intramuscularly did not show any visible reaction, but in accordance with these authors, the piglets from the present study did not present any abscess-like reaction 21 days post-vaccination.

## Conclusions

It is concluded that Hipradermic needle-free vaccination may be less painful than is intramuscular vaccination for piglets because animals showed a lower prevalence of vocalizations, including those of more than 1,000 Hz, and a lower level of pressure of sound (dB). In addition, piglets demonstrated more signs of aversion when vaccinated intramuscularly (with 2.0 ml of UNISTRAIN®) than intradermally without a needle (with 0.2 ml of UNISTRAIN®) as evidenced by the highest times in crossing the raceway 10 min after vaccination during the aversion test. In addition, this effect disappeared at 2 h after the vaccination, so the aversion should be considered of low severity. Both types of vaccination induced some behavioral changes, such as lying on the side of the vaccination a few hours after the administration, and skin reactions, such as papules and blood spots in the first minutes, and crusts and redness areas 8 h after the administration. Intramuscular and intradermal routes of vaccine administration produced good immunity against the PRRS. In terms of animal welfare, needle-free vaccination should be promoted in the future for piglets.

## Data Availability Statement

The raw data supporting the conclusions of this article will be made available by the authors, without undue reservation.

## Ethics Statement

The animal study was reviewed and approved by Generalitat de Catalunya Departament de Territori i Sostenibilitat Direcció General de Polítiques Ambientals i Medi Natural under code 11026.

## Author Contributions

AD was the coordinator of the study and responsible for writing the paper. AS-M and JM were involved in the conception and design of the study. AX and AV-P were responsible for performing the aversion tests, vaccinating the animals, and assessing the reactions to the vaccination. IM was responsible for analyzing the vocalizations. XM and BB were responsible for recording the vocalizations, socialization of piglets, and taking blood samples. EF and JP were responsible for saliva sampling. AV and AC-J were responsible for performing and analyzing the activity budgets. AP was responsible for vaccination training and management. AC-J was, as well, a co-coordinator of the study and co-responsible for writing the paper, although all authors checked the manuscript until their final approval.

## Conflict of Interest

AS-M, JM, and AP are employees of the Laboratorios HIPRA SLU. AS-M and JM were involved in the conception and design of the study. This means that they asked to the IRTA researchers what they wanted to study and IRTA researchers proposed how and when. They did as well some amendments to the firsts drafts of the protocol according to the best practices of laboratory and IRTA researchers considered those that were improving the methodology. The case of AP was different because she was responsible of training AX and AV-P in vaccinating the animals properly, to provide the vials for vaccination, and to check for the company that the day of the vaccination everything was done according to the protocol. None of these three persons had access to the animals for any of the parameters assessed neither to the data. This paper comes from the report that IRTA sent to this company at the end of the study. The remaining authors declare that the research was conducted in the absence of any commercial or financial relationships that could be construed as a potential conflict of interest.

## Publisher's Note

All claims expressed in this article are solely those of the authors and do not necessarily represent those of their affiliated organizations, or those of the publisher, the editors and the reviewers. Any product that may be evaluated in this article, or claim that may be made by its manufacturer, is not guaranteed or endorsed by the publisher.
